# TULA-2 Deficiency Enhances Platelet Functional Responses to CLEC-2 Agonists

**DOI:** 10.1055/s-0038-1676358

**Published:** 2018-12-05

**Authors:** John C. Kostyak, Benjamin R. Mauri, Carol Dangelmaier, Akruti Patel, Yuhang Zhou, Johannes A. Eble, Alexander Y. Tsygankov, Steven E. McKenzie, Satya P. Kunapuli

**Affiliations:** 1 Sol Sherry Thrombosis Research Center, Lewis Katz School of Medicine, Temple University, Philadelphia, Pennsylvania, United States; 2 Cardeza Foundation for Hematologic Research, Department of Medicine, Thomas Jefferson University, Philadelphia, Pennsylvania, United States; 3 Institute of Physiological Chemistry and Pathobiochemistry, University of Munster, Waldeyerstasse, Munster, Germany; 4 Department of Immunology and Microbiology, Lewis Katz School of Medicine, Temple University, Philadelphia, Pennsylvania, United States

**Keywords:** platelets, thrombosis, thromboxane A _2_

## Abstract

Platelet activation is essential for hemostasis. Central to platelet activation are the signals transmitted through surface receptors such as glycoprotein VI, the protease-activated receptors, and C-type lectin-like receptor 2 (CLEC-2). CLEC-2 is a HemITAM (hem-immunoreceptor tyrosine activation motif)-bearing receptor that binds podoplanin and signals through spleen tyrosine kinase (Syk). T-cell ubiquitin ligand-2 (TULA-2) is a protein tyrosine phosphatase that is highly expressed in platelets and targets phosphorylated Y352 of Syk. We wanted to determine whether TULA-2 regulates Syk phosphorylation and activity downstream of CLEC-2. To that end, we used TULA-2 knockout mice and wild-type (WT) littermate controls. We found that TULA-2 deficiency enhances the aggregation and secretion response following stimulation with an excitatory CLEC-2 antibody or the CLEC-2 agonist rhodocytin. Consistently, Syk phosphorylation of Y346 is enhanced, as well as phosphorylation of the downstream signaling molecule PLCγ2, in TULA-2 knockout platelets treated with either CLEC-2 antibody or rhodocytin, compared with WT control platelets. Furthermore, the kinetics of Syk phosphorylation, as well as that of PLCγ2 and SLP-76, is enhanced in TULA-2 knockout platelets treated with 2.5-μg/mL CLEC-2 antibody compared with WT platelets. Similarly, thromboxane production was enhanced, in both amount and kinetics, in TULA-2 ^−/−^ platelets treated with 2.5-μg/mL CLEC-2 antibody. TULA-2 acts as a negative regulator of CLEC-2 signaling by dephosphorylating Syk on Y346 and restraining subsequent Syk-mediated signaling.

## Introduction

Platelets are the primary mediators of thrombosis and hemostasis. They are small anucleate cells that exist in a quiescent state, and in a discoid shape. Platelets respond to vascular damage by binding to von Willebrand factor and collagen, which initiates an intracellular signaling cascade that culminates in shape change, production of thromboxane A _2_ (TXA), and release of granular contents. Release of secondary mediators such as TXA and adenosine diphosphate reinforces the original excitatory signal and recruits new platelets to the growing thrombus. Just as this process is essential to maintain hemostasis, a similar process is necessary to insure lymphatic and blood vessel separation.

Lymphatic endothelial cells differentiate from venous endothelial cells and, unlike venous and arterial endothelial cells, they express podoplanin, which is the only known physiologic ligand for C-type lectin-like receptor 2 (CLEC-2). 1 2 3 CLEC-2 is highly expressed on platelets and megakaryocytes and is a hem-immunoreceptor tyrosine activation motif (hemITAM) containing receptor, which means it has one part of an ITAM motif (Y *XX* (L/I)). The binding of podoplanin to CLEC-2 initiates a signaling cascade that involves tyrosine phosphorylation of the hemITAM via Src-family kinases (SFKs), and subsequent spleen tyrosine kinase (Syk) phosphorylation. 4 Initiation of this signaling cascade results in platelet activation.

Platelets, along with lymphatic endothelial cells, are responsible for maintaining separation of blood and lymph. Deletion of either SH2 domain–containing leukocyte protein of 76 kDa (SLP-76) or Syk (crucial for signaling via CLEC-2) in mice resulted in embryonic lethality due to disrupted separation of blood and lymph. 5 Furthermore, deletion of either CLEC-2 or podoplanin resulted in the mixing of blood and lymphatics. 6 7 8 Indeed, platelet-specific conditional knockout mice of either CLEC-2, Syk, or SLP-76 had similar phenotypes to the corresponding global knockout model, suggesting that platelet CLEC-2 is essential for proper separation of blood and lymph. 9 10 The above data demonstrate that it is CLEC-2 on the platelet surface binding to podoplanin on the surface of lymphatic endothelial cells that is responsible for the initial platelet activation necessary to create microthrombi that maintain separation of blood and lymphatic fluid. 11

The CLEC-2 and podoplanin are also important for several other physiological and pathological processes. There is evidence in the literature that CLEC-2 plays a role in thrombosis and hemostasis, probably in conjunction with glycoprotein VI (GPVI) as deletion of both CLEC-2 and GPVI in mice causes bleeding and reduced arterial thrombus formation. 12 Furthermore, CLEC-2 is known to promote hematogenous tumor metastasis of podoplanin-expressing cells. 13 Finally, high podoplanin expression in primary brain tumors is associated with an increased risk of venous thromboembolism, due to platelet activation via the CLEC-2/podoplanin interaction. 14

Syk is a crucial mediator of signaling initiated by podoplanin engagement of CLEC-2. Syk is phosphorylated on several tyrosine residues following activation of CLEC-2, just as it is following activation of GPVI, which contains two ITAM motifs. 15 16 Of particular importance to the work presented in this paper is Y346, which, when phosphorylated with Y342, acts to prevent Syk folding into an autoinhibited conformation, and Y519/520, which is located in the activation loop of Syk and is used as a marker for Syk activity. 17 18 19 20 Phosphorylation of all residues mentioned above is thought to be required for full activation of Syk. 21 In addition to phosphorylation following hemITAM or ITAM activation, dephosphorylation via phosphatase activity also contributes to the regulation of Syk.

Syk is a target of the protein tyrosine phosphatase T-cell ubiquitin ligand-2 (TULA-2). 22 23 24 TULA-2 has been reported to function in a variety of cell types including platelets. 22 23 25 26 27 28 29 30 We have previously shown that TULA-2 deficiency enhances thrombosis, while TULA-2 overexpression protects mice against thrombosis. 29 31 Furthermore, we revealed that the target of TULA-2 is Syk Y346 (Y352 in human), the phosphorylation of which, in concert with Y342 (Y348 in human), acts to prevent Syk from adopting an autoinhibitory conformation. 22 32 33 34 While this appears to be the case downstream of the ITAM-containing receptors GPVI and FcγRIIA, the function of TULA-2 had not been elucidated downstream of the hemITAM-containing receptor CLEC-2. 22 35 Therefore, we used TULA-2-deficient mice to explore the function of TULA-2 in platelet reactivity following stimulation of the CLEC-2 receptor.

## Materials and Methods

### Antibodies and Reagents

All reagents were purchased from Thermo Fischer Scientific unless otherwise noted. Rhodocytin was isolated as previously described. 36 Chronolume, used for the detection of secreted adenosine triphosphate (ATP), was purchased from Chrono-log Corp. (Havertown, PA). The CLEC-2-activating antibody (17D/CLEC-2) was purchased from Biolegend (San Diego, CA), while donkey anti-rat (DAR) IgG was purchased from Novex (Frederick, MD). Anti-pSyk Y352, anti-pSyk Y525/Y526, anti-pPLCγ2 Y759, anti-pPLCγ2 Y1217, and anti-SLP76 were all purchased from Cell Signaling Technology (Beverly, MA). Please note that Syk pY sites listed correspond to human Syk as listed by the manufacturer. The numbering of mouse pY sites is different but will be noted in the paper. Anti-Syk and anti-PLCγ2 were purchased from Santa Cruz Biotechnology (Santa Cruz, CA). Anti-pSLP-76 Y128 was purchased from BD Transduction (San Jose, CA). Odyssey blocking buffer and secondary antibodies IRDye 800CW goat anti-rabbit and IRDye 680LT goat anti-mouse were purchased from Li-Cor (Lincoln, NE). Please see the “major resources” table for further antibody information.

### Mice

TULA-2 knockout mice were described previously. 26 All work involving mice was done according to the Temple University Institution Animal Care and Use Committee. All mice were housed in a pathogen-free environment of the Temple University Central Animal Facility. Please see the “major resources” table for more information.

### Isolation of Murine Platelets

Mouse blood was collected and processed as previously described. 22 Briefly, blood from anesthetized mice was collected via cardiac puncture into one-tenth volume 3.8% sodium citrate and centrifuged at 100 × g for 10 minutes. The platelet-rich plasma (PRP) was removed and sodium citrate was added to the remaining packed cells prior to a second 100 × g spin. The resulting PRPs were combined and 1 μM PGE1 was added to the PRP before centrifugation at 400 × g for 10 minutes. The resulting platelet pellet was resuspended in Tyrodes buffer (138-mM NaCl, 2.7-mM KCl, 2-mM MgCl _2_ , 0.42-mM NaH _2_ PO _4_ , 10-mM HEPES, and 0.2-U/mL apyrase, pH 7.4), and platelets were counted using a Hemavet 950FS blood cell counter (Drew Scientific, Dallas, TX) and adjusted to a final concentration of 1.5 × 10 ^8^ cells/mL.

### Platelet Aggregation and ATP Secretion

All aggregation and secretion experiments were performed using a lumi-aggregometer (Chrono-log) at 37°C under stirring conditions. Platelets (250 μL) were stimulated with rhodocytin, CLEC-2 antibody, or CLEC-2 antibody plus DAR-IgG and aggregation was measured via light transmission. ATP secretion was measured simultaneously using Chrono-lume (a luciferine/luciferase reagent).

### Thromboxane Generation

Washed murine platelets (50 μL) isolated from TULA-2 ^−/−^ and wild-type (WT) mice were heated to 37°C without stirring. The platelets were then stimulated with 2.5 μg/mL of CLEC-2 antibody and allowed to incubate at 37°C. The reaction was stopped by snap freezing. Samples were collected every 30 seconds for 6 minutes. Thromboxane B _2_ (a stable analogue of TXA) was measured by enzyme-linked immunosorbent assay (ELISA), using a kit from Enzo Life Sciences (Farmingdale, NY) as per the manufacturer's instructions.

### Western Blotting

Western blotting was performed as described previously. 22 Briefly, platelets were stimulated for the appropriate time points with 2.5 mg/mL CLEC-2 antibody at 37°C under stirring conditions. Platelet proteins were precipitated using 0.6 N HClO _4_ and washed with water prior to solubilization in sample buffer. Platelet protein samples were then boiled for 5 minutes. Proteins were resolved via SDS-PAGE and transferred to nitrocellulose membranes. The membranes were blocked with Odyssey blocking buffer and incubated overnight with primary antibodies against a protein of interest. After washing, the membranes were incubated with the appropriate secondary antibodies for 1 hour at room temperature. The membranes were then washed and imaged using a Li-Cor Odyssey infrared imaging system.

### Statistics

All statistical comparisons were calculated using Student's *t* -test. Significance was determined by *p* < 0.05. All bar graphs represent mean ± SEM for that dataset.

## Results

### TULA-2 Deficiency Enhances Platelet Aggregation and Secretion Following Stimulation of the CLEC-2 Receptor

**Fig. 1:**
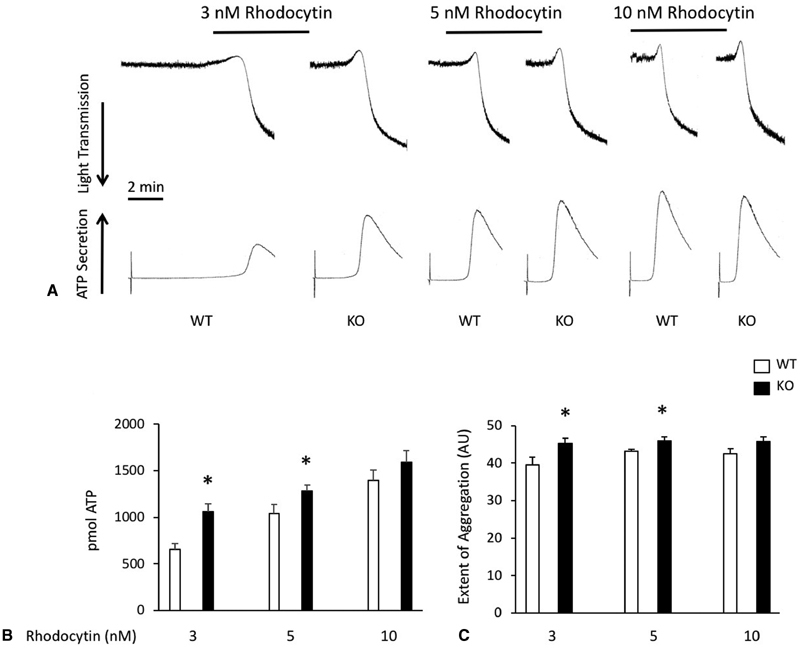
TULA-2 deficiency enhances the platelet response to the CLEC-2 agonist rhodocytin. **(A)** Platelets from WT and TULA-2 ^−/−^ (knockout) mice were stimulated with the indicated concentrations of rhodocytin, and aggregation and ATP secretion were recorded. **(B)** Quantification (mean ± SEM) of ATP secretion from WT and TULA-2 ^−/−^ murine platelets stimulated with the indicated concentrations of rhodocytin. **(C)** Quantification (mean ± SEM) of the extent of aggregation expressed as arbitrary units. * *p* < 0.05, *n* = 5.

Rhodocytin is a snake venom protein that binds and activates the CLEC-2 receptor. 7 36 37 In response to low doses of rhodocytin, platelets from TULA-2 knockout mice responded more robustly than WT control platelets ( Fig. 1 ). The kinetics of aggregation and secretion are both enhanced at 3 and 5 nM rhodocytin in TULA-2 null platelets compared with WT platelets ( Fig. 1A ). Similarly, the amount of secreted ATP and the extent of aggregation were higher in platelets from TULA-2 mice compared with platelets from WT mice at those concentrations ( Fig. 1B, C ). No differences were noted when using higher concentrations of rhodocytin (10 nM).

**Fig. 2:**
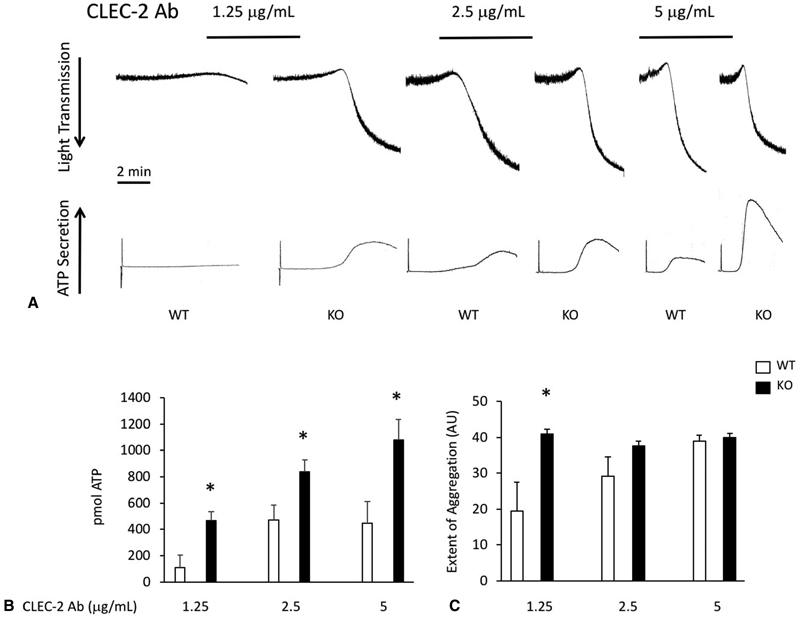
Platelet functional responses to a CLEC-2 antibody are greatly enhanced in the absence of TULA-2. **(A)** Platelets from WT and TULA-2 ^−/−^ (knockout) mice were stimulated with the indicated concentrations of an antibody against mouse CLEC-2 and aggregation and secretion responses were recorded. **(B)** Quantification (mean ± SEM) of secreted ATP at the indicated concentrations of CLEC-2 antibody. **(C)** Quantification (mean ± SEM) of the extent of aggregation expressed as arbitrary units. * *p* < 0.05, *n* = 5.

To confirm the data generated using rhodocytin, we utilized a CLEC-2-activating antibody and performed aggregation and secretion experiments using platelets from TULA-2 null mice. Using any concentration of CLEC-2 antibody from 1.25 to 5 μg/mL, we observed an enhanced response from TULA-2 ^−/−^ platelets compared with WT control platelets ( Fig. 2A , B ). This was characterized by an increase in the kinetics of aggregation and secretion, as well as the amount of secreted ATP. The extent of aggregation was also enhanced at the lowest concentration of CLEC-2 antibody used ( Fig. 2C ). Together with the data presented in Fig. 1 , these data suggest that TULA-2 is an important mediator of platelet reactivity downstream of the CLEC-2 receptor.

**Fig. 3:**
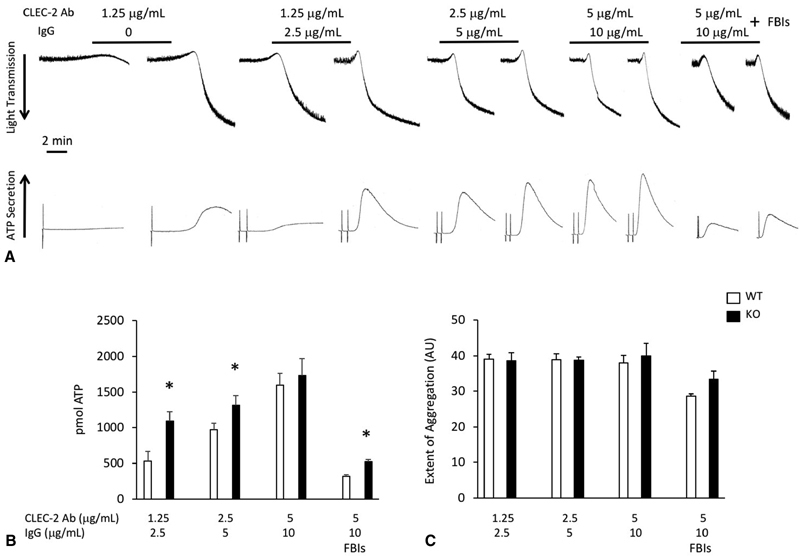
Platelet response to CLEC-2 double crosslinking is enhanced with TULA-2 deficiency. **(A)** Platelets from WT and TULA-2 ^−/−^ (knockout) mice were stimulated with the indicated concentrations of CLEC-2 antibody for 30 seconds, after which a donkey anti-rat secondary antibody was added to promote crosslinking. Aggregation and secretion responses were then recorded. The 1.25-μg/mL CLEC-2 antibody representative panel from Fig. 2 is included for reference. FBI, feedback inhibitors, which consist of 10-μM MRS-2179, 100-nM AR-C69931MX, and 10-μM indomethacin. **(B)** Quantification (mean ± SEM) of ATP secretion from WT and TULA-2 ^−/−^ platelets stimulated with CLEC-2 antibody and donkey anti-rat secondary antibody. **(C)** Quantification (mean ± SEM) of the extent of aggregation expressed as arbitrary units. * *p* < 0.05, *n* = 5.

Both rhodocytin and the CLEC-2 antibody activate CLEC-2 via clustering. 36 37 It has also been proposed that clustering of CLEC-2 and its physiological ligand podoplanin is important for platelet adhesion to the lymphatic endothelium and the subsequent separation of blood and lymph. 4 Therefore, we used a secondary antibody (IgG) in addition to the primary CLEC-2 antibody to enhance CLEC-2 receptor crosslinking. Addition of 2.5 and 5 μg/mL IgG to 1.25 and 2.5 μg/mL CLEC-2 antibody, respectively, increased the response observed with CLEC-2 antibody alone in both WT and TULA-2 ^−/−^ platelets ( Fig. 3A , B ). However, secretion was enhanced in TULA-2 ^−/−^ platelets compared with WT platelets ( Fig. 3A , B ). No differences in aggregation were noted ( Fig. 3C ). Furthermore, while the addition of 10 μg/mL IgG to 5 μg/mL CLEC-2 antibody did enhance the aggregation and secretion response in both WT and TULA-2 ^−/−^ platelets compared with CLEC-2 antibody alone, there was no difference in either metric noted between the two groups ( Fig. 3A , B ). To determine whether TULA-2 regulates primary signaling from the CLEC-2 receptor, we preincubated WT and TULA-2 ^−/−^ platelets with 10-μM MRS-2179 to antagonize the P2Y1 receptor, 100-nM AR-C69931MX to antagonize the P2Y12 receptor, and 10-μM indomethacin to inhibit thromboxane production prior to stimulation with 5-μg/mL CLEC-2 antibody and 10-μg/mL IgG. Both aggregation and secretion are enhanced in TULA-2 ^−/−^ platelets compared with WT platelets in this scenario, suggesting that TULA-2 negatively regulated primary CLEC-2 signaling. These data suggest that clustering enhances the CLEC-2-mediated aggregation and secretion response and that TULA-2 is a negative regulator of that response.

### Thromboxane Generation Is Enhanced in TULA-2 Knockout Platelets Stimulated with a CLEC-2 Antibody

**Fig. 4:**
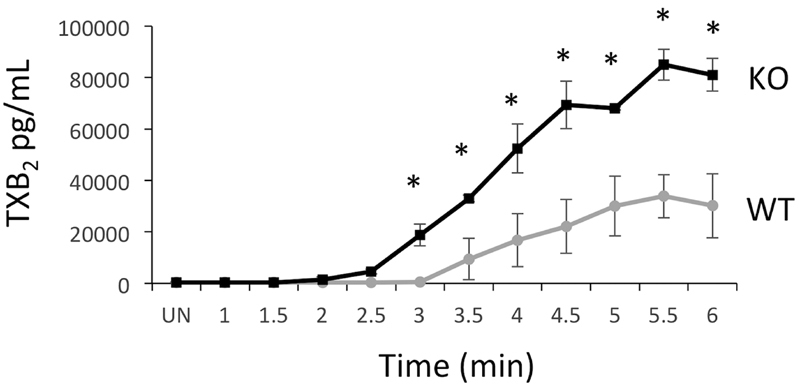
Thromboxane production is enhanced in TULA-2 ^−/−^ platelets compared with WT control platelets. Platelets from WT and TULA-2 ^−/−^ (knockout) mice were stimulated with 2.5 μg/mL of CLEC-2 antibody and incubated at 37°C. Samples were collected by snap freezing every 30 seconds for a period of 6 minutes. Data expressed as mean ± SEM. * *p* < 0.05, *n* = 6.

Generation of thromboxane is an important facet of platelet activation. Because aggregation and secretion were enhanced in TULA-2 ^−/−^ platelets, we hypothesized that thromboxane generation would be similarly enhanced. To determine whether there were differences in the kinetics of thromboxane generation, maximal thromboxane generation, or both, we stimulated WT and TULA-2 knockout platelets with 2.5-μg/mL CLEC-2 antibody at 37°C for up to 6 minutes without stirring. Under these conditions, we were able to detect thromboxane generation 2.5 minutes after CLEC-2 stimulation in TULA-2 ^−/−^ platelets, but were not able to detect any thromboxane until 3.5 minutes after stimulation in WT platelets ( Fig. 4 ). Furthermore, peak thromboxane production was nearly threefold greater in TULA-2 ^−/−^ platelets. These data suggest that the kinetics of thromboxane production and the amount of thromboxane produced is greatly elevated in TULA-2 ^−/−^ platelets compared with WT platelets following stimulation of CLEC-2.

### Phosphorylation of Syk Is Enhanced in TULA-2 ^−/−^ Platelets Following Stimulation of CLEC-2

**Fig. 5:**
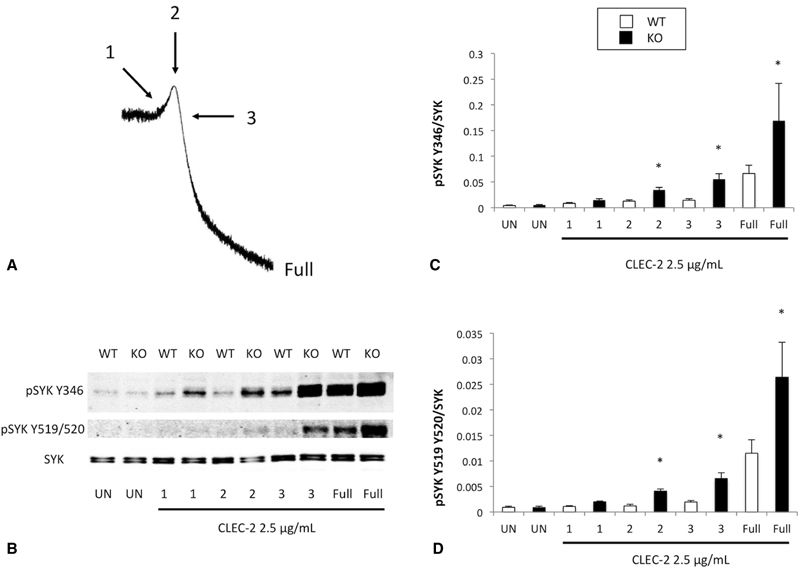
Syk phosphorylation is enhanced in TULA-2 ^−/−^ platelets after CLEC-2 stimulation. **(A)** Schematic showing the time points used for protein precipitation following CLEC-2 stimulation. A side-by-side comparison of WT and TULA-2 ^−/−^ platelet aggregation following 2.5-μg/mL CLEC-2 antibody stimulation can be found in Fig. 2 . **(B)** Representative western blots showing pSyk Y346, pSyk Y519/520, and total Syk in WT and TULA-2 ^−/−^ (knockout) platelets stimulated with a CLEC-2 antibody for the indicated time points (UN = unstimulated). **(C, D)** Quantification (mean ± SEM) of band intensities from several independent experiments represented in ( **A** ) expressed as a ratio of phosphorylated Syk to total Syk. * *p* < 0.05, *n* = 5.

Syk is an important signaling molecule that is phosphorylated downstream of the CLEC-2 receptor and regulated by TULA-2. 15 29 Therefore, we sought to analyze the phosphorylation status of Syk following CLEC-2 stimulation in WT and TULA-2 ^−/−^ platelets. The residues Y346 and Y519/Y520 are involved in the regulation of Syk activity. We performed time course experiments using 2.5 μg/mL of the CLEC-2 antibody as an agonist and evaluated the phosphorylation of Y346 and Y519/520 on Syk. Four time points were collected in each experiment and compared with unstimulated (UN) samples. The time points are presented in Fig. 5A . Each point was collected first in TULA-2 ^−/−^ samples and the time to reach that point was recorded. That time was used to collect the complementary point from WT platelets. For instance, if point 1 (the beginning of shape change) was reached in 30 seconds using TULA-2 ^−/−^ platelets, then the complementary WT sample was collected 30 seconds after agonist stimulation. We also collected a sample when maximal aggregation was reached, which we termed “full.” Following SDS-PAGE of the samples collected using the previously described method, it was clear that Syk Y346 and Y519/520 were phosphorylated earlier and to a greater extent in TULA-2 ^−/−^ platelets than WT platelets after stimulation with a CLEC-2-activating antibody ( Fig. 5B ). Analysis of band intensity revealed significant increases in phosphorylation of each residue at time points 2 and 3, as well as following full aggregation ( Fig. 5C , D ). These data suggest that Syk phosphorylation is enhanced downstream of the CLEC-2 receptor in TULA-2 ^−/−^ platelets.

**Fig. 6:**
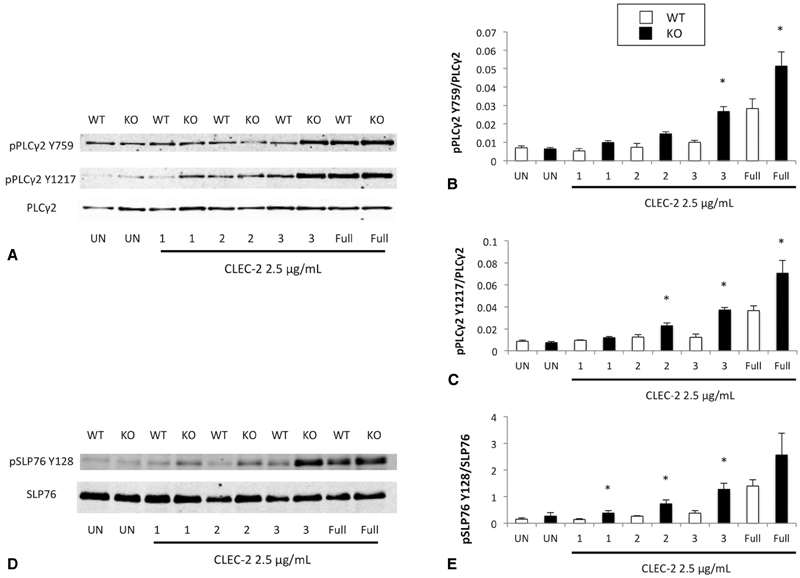
Phosphorylation of Syk substrates is enhanced in TULA-2 ^−/−^ mice. **(A)** Representative western blots showing pPLCγ2 Y759, pPLCγ2 Y1217, and total PLCγ2 in platelet preparations from WT and TULA-2 ^−/−^ (knockout) mice stimulated with 2.5-μg/mL CLEC-2 antibody. Time points collected are the same as those described in Fig. 5 (UN = unstimulated). **(B, C)** Quantification (mean ± SEM) of band intensities from the experiments represented in ( **A** ). **(D)** Representative western blot depicting SLP-76 Y128 and total SLP-76 prepared from WT and TULA2 ^−/−^ platelets. **(E)** Quantification (mean ± SEM) of band intensities from experiments represented in ( **D** ). * *p* < 0.05, *n* = 5.

Phospholipase Cγ2 (PLCγ2) and SLP-76 are both phosphorylated downstream of Syk. Therefore, we analyzed the phosphorylation of PLCγ2 and SLP-76 using the same methodology described above. We observed that PLCγ2 was phosphorylated earlier and to a greater extent at both Y759 and Y1217 in TULA-2 ^−/−^ platelets compared with WT platelets, similar to the pattern observed in our analysis of Syk phosphorylation ( Fig. 6A ) following stimulation of CLEC-2. Quantification of band intensities supported this assertion ( Fig. 6C , D ). Additionally, we determined the phosphorylation state of SLP-76 on Y128. Similar to Syk and PLCγ2, phosphorylation of SLP-76 occurred earlier in TULA-2 knockout platelets. However, there was no significant difference in SLP-76 phosphorylation in samples collected after maximal aggregation was achieved. These data support the notion that TULA-2 regulates Syk downstream of CLEC-2, as the phosphorylation of two known proteins downstream of Syk activation follows a similar pattern to that of Syk using TULA-2 null platelets.

## Discussion

In this report, we demonstrate that TULA-2 is a negative regulator of signaling initiated by the hemITAM-containing CLEC-2 receptor. The regulation of CLEC-2-induced signaling may be important for several physiological processes such as separation of blood and lymph, thrombosis and hemostasis, and tumor cell metastasis. 11 12 13 CLEC-2 signaling is dependent on Syk. Therefore, a better understanding of Syk regulation via a phosphatase such as TULA-2 allows for improved understanding of the above processes.

In terms of Syk phosphorylation, our data agree with published data describing the effect of TULA-2 on Syk downstream of two ITAM-containing receptors, FcγRIIA and GPVI. 22 35 Similarly, aggregation and secretion were also enhanced using TULA-2 ^−/−^ mouse platelets compared with WT control platelets when either GPVI or FcγRIIA were stimulated. This suggests that TULA-2 acts as an important regulator of Syk phosphorylation when Syk is activated by either a hemITAM or ITAM-containing receptor. Upon ligand engagement of a hemITAM or ITAM-containing receptor, SFKs phosphorylate the tyrosine within the YXX(L/I) motif (one tyrosine per hemITAM and 2 per ITAM). 38 39 Syk can then dock via its tandem SH2 domains, at which time it undergoes autophosphorylation. Phosphorylation at Y342 and Y346 prevents Syk from refolding into its inhibitory conformation. 34 Furthermore, phosphorylation of Y342 and Y346 not only is important for Syk activity, but also influences the phosphorylation of Y519/520. 29 32 33 40 Our data support this model as phosphorylation of Y346 and Y519/520 is enhanced in TULA-2 ^−/−^ platelets following stimulation of CLEC-2.

Our data show that Syk phosphorylation not only is greater in TULA-2 ^−/−^ platelets stimulated with a CLEC-2 antibody, but also is detected earlier than similarly treated WT platelets. Furthermore, Syk phosphorylation continues to persist, and even increases throughout the aggregation process, which does not occur in time course experiments when platelets are stimulated with agonists to GPVI. 22 In the case of GPVI stimulation, Syk Y346 phosphorylation occurs within 30 seconds in both WT and TULA-2 ^−/−^ platelets. Interestingly, Syk Y346 and Y519/520 phosphorylation decreases from that point forward, even in TULA-2 ^−/−^ platelets.

The reason for this discrepancy could be that a burst of Syk phosphorylation occurs following GPVI stimulation due to the ITAM having two YXX(L/I) motifs, which would allow Syk to bind immediately upon GPVI activation, thus resulting in phosphorylation of a large portion of total Syk. From that point, any alterations in Syk phosphorylation could be due to phosphatase activity. The absence of a phosphatase such as TULA-2 would delay the reduction in Syk phosphorylation, at least at Y346. Conversely, time may be required for CLEC-2 to cluster and present two hemITAM domains for Syk to bind (one for each tandem SH2 domain) and autophosphorylate, which would delay Syk phosphorylation. The absence of a phosphatase such as TULA-2 to remove the initial phosphorylation on the small amount of Syk that is phosphorylated initially may give the impression that Syk is phosphorylated at an earlier time point when, in fact, it is not dephosphorylated. Furthermore, CLEC-2 clustering due to the CLEC-2 antibody may continue throughout the aggregation process leading to greater Syk phosphorylation as the experiment continues. The lack of TULA-2 would only enhance the increase in Syk phosphorylation, which is exactly what we observed.

It is also possible that the phosphatases that function downstream of GPVI are different from those that function downstream of CLEC-2. The data reported for Syk phosphorylation downstream of GPVI in the absence of TULA-2 suggest that there are phosphatases other than TULA-2 that dephosphorylate Syk, as Syk phosphorylation is reduced over time. Our data suggest that TULA-2 could be the main initial phosphatase that acts on Syk downstream of CLEC-2, since Syk phosphorylation continues to increase over time in the absence of TULA-2.

We have previously detailed an important crosstalk mechanism that occurs between CLEC-2 and G _q_ . 41 Syk phosphorylation is enhanced when the thromboxane mimetic U46619 is added to platelets stimulated with rhodocytin, but no Syk phosphorylation is caused by U46619 alone. The data suggest that signaling through the TP receptor, which is coupled to G _q_ and G _12/13_ , can augment Syk phosphorylation when CLEC-2 is likewise stimulated. In this report we demonstrated that thromboxane production is greatly enhanced in TULA-2 ^−/−^ platelets stimulated with a CLEC-2 antibody compared with WT control platelets. Therefore, it is possible that the enhanced Syk phosphorylation observed downstream of the CLEC-2 receptor in TULA-2 ^−/−^ platelets is partly due to feedback from the TP receptor.

We demonstrate in this report that TULA-2 acts as a negative regulator of CLEC-2-mediated signaling by dephosphorylating its target substrate, Syk. TULA-2 is one of several phosphatases, such as SHP-1, SHP-2, SHIP-1, and CD148, that control ITAM and hemITAM-mediated signaling in platelets. 42 43 44 There is likely functional redundancy between some of these phosphatases (i.e., those that dephosphorylate SFKs would also effect Syk). Regardless, we show that platelet responses to CLEC-2 agonists are enhanced in the absence of TULA-2 due to enhanced signaling characterized by increased Syk phosphorylation and subsequent thromboxane production.
